# The Effect of Perceived Over-Qualification on Turnover Intention From a Cognition Perspective

**DOI:** 10.3389/fpsyg.2021.699715

**Published:** 2021-10-15

**Authors:** Guofu Chen, Yanzhao Tang, Yawen Su

**Affiliations:** ^1^School of Management, Xiamen University, Xiamen, China; ^2^School of Education, The Education University of Hong Kong, Tai Po, Hong Kong, SAR China

**Keywords:** perceived over-qualification, turnover intention, self-efficacy, professional identity, elementary school teachers, cognition

## Abstract

Employee turnover caused by over-qualification has become a new problem in organizational management. The mechanism underpinning the boundaries between perceived over-qualification and employee turnover, however, remains unclear. To address this gap, the current study employed multi-factor ANOVA, hierarchical regression analysis and the bootstrap method to analyze the relationship between perceived over-qualification and employee turnover intention based on the survey data of 396 respondents in China. Overall, the results revealed that perceived over-qualification was positively correlated with turnover intention. It was also found that self-efficacy had a mediating effect on the relationship between perceived over-qualification and turnover intention. Further, professional identity had a moderating effect on the relationship between perceived over-qualification and turnover intention. Our findings expand the boundary of influence around perceived over-qualification and provide theoretical support for employee management.

## Introduction

Over-qualification refers to a situation in which an individual’s education level, work experience and ability exceed their work needs (McKee-Ryan and Harvey, 2011; Liu and Mo, 2012). Two paths can be considered when investigating the influence of perceived over-qualification on individual behavior: the emotional path and the cognitive path (Harari et al., 2017; Cheng et al., 2019; Zhao C. H. et al., 2019). For example, Cheng et al. (2019) explored the effect of perceived over-qualification on career satisfaction based on the cognitive path. Most existing studies predominantly focus on the influence of perceived over-qualification on employees’ positive behavior (Erdogan and Bauer, 2009; Yang et al., 2015; Sesen and Ertan, 2019), while studies on the influence of perceived over-qualification on employees’ negative emotions and behaviors are scant. Employee’ perceived over-qualification may negatively impact organizations and co-workers, such as through deliberately going slow, anti-productive behavior, and turnover intention (Zhang et al., 2016; Lin et al., 2017).

Employee turnover is an important issue in organizational management and can be divided into turnover intention and turnover behavior (Weng and Xi, 2010; Memon et al., 2019; Baranchenko et al., 2020). In comparison to turnover behavior, turnover intention relates to individual willingness. Existing studies on turnover predominantly focus on the abovementioned two aspects of it (turnover intention and turnover behavior), and findings suggest turnover intention can lead to employees’ turnover behavior (Allen et al., 2005; Cho and Lewis, 2012). Specifically, an employee’s psychological intention to leave an organization will lead to the behavior of terminating their relationship with the organization. Employees’ turnover intention is related to their level of dissatisfaction due to various reasons and may lead to resignation (Hancock et al., 2013). Most studies on the factors underpinning turnover intention focus on personal age, health and wellbeing, work environment, workload, and the welfare and wages (Daouk et al., 2013; Wang et al., 2019). Additionally, quality of life and job burnout may affect employees’ turnover intention (Hayes et al., 2012). Job satisfaction is also thought to reduce employees’ turnover intention (Galletta et al., 2011; Chen et al., 2017; Suifan et al., 2017; Lee, 2019). In-depth research on employee turnover intention is helpful in exploring the formation process underpinning such intention. This type of research is also conducive to determining the defensive measures necessary for organizations to improve their management.

Turnover intention is the combination of employees’ intention to leave, seek other employment, and the possibility they will find another job (Zhang W. et al., 2018). When an employee’s original job does not satisfy their needs or prevents them from realizing their value, they may develop the turnover intention to pursue better career development opportunities toward helping them realize this value. According to the “person-job” matching theory (Mobley et al., 1978), when personal characteristics are inconsistent with the professional environment, work efficiency is significantly reduced (Xie et al., 2015; Ma et al., 2021). People’s personality characteristics and the professional environment in which they work need to align within a certain matching range. A significant mismatch between these two factors can lead to problems such as deliberately going slow and turnover behavior (Tims et al., 2016; Ding et al., 2019). Therefore, measuring employee’ perceived over-qualification can help organizations anticipate problems, in turn reducing employees’ negative emotions and turnover intention.

At present, there are only a few studies that focus on the exploration of the relationship between perceived over-qualification and turnover intention. For example, Cuyper et al. (2011) pointed out that perceived employ ability was positively related to turnover intention. Maynard et al. (2006) indicated that perceived over-qualification was also an antecedent variable of turnover behavior. Triana et al. (2017) found that perceived over-qualification moderates the effect of perceived age discrimination on job withdrawal. Although Wang et al. (2019) analyzed the relationship between perceived over-qualification and turnover intention, the study did not elaborate on the specific influence path. Based on the literature review conducted for the current study, it was found that the mechanism underpinning the boundary conditions between over-qualification and employees’ negative behavior remains unclear (Harari et al., 2017). There remains a significant gap in the current theoretical research on perceived over-qualification and its relationship with turnover intention, particularly in terms of the type of influence path that is implicated.

In addition, existing studies mostly focus on corporate employees. One possible reason is that perceived over-qualification of corporate employees is rather different and their turnover behavior is more frequent. Second, the number of corporate employees is large and universal, which is conducive to investigation and data collection. However, as the cost of job change for corporate employees is relatively small, many corporate employees with high qualifications tend to choose better jobs. Therefore, corporate employee’ perceived over-qualification is relatively low. In the current study, the main focus is enterprise employees, which ignores the problem of over-qualification of institution establishment worker. With continuous improvement in employees’ recruitment thresholds and increases in employee turnover within the institutional system, more attention needs to be paid to the problem of employee over-qualification and turnover in institutional system (Verhaest and Omey, 2006). The level of employee’ perceived over-qualification in the institutional system is relatively high, but due to the high cost of job change (among other reasons), employees often feel “over-capable,” are more likely to produce negative emotions and anti-productive behaviors, and may even harbor high turnover intentions (Maynard et al., 2006; Zhao L. J. et al., 2019). Currently, it is not uncommon for highly-educated and highly-qualified graduates from top schools to teach in primary and secondary schools, which has generated a heated discussion around the “over-capability of underachievers.” The reasons behind this and the impact it causes deserve attention. Meanwhile, this phenomenon also allows for reflection on the problem of over-qualification among elementary school teachers.

Based on the literature review, the current study selected primary school teachers as participants to explore the relationship between perceived over-qualification and turnover intention, as well as the mediating role of the cognitive pathway. The current study predominantly focuses on four components: (1) the differential performance of employee’ perceived over-qualification; (2) the relationship between perceived over-qualification and turnover intention; (3) the mediating effect of self-efficacy on the relationship between perceived over-qualification and turnover intention; and (4) the moderating effect of professional identity on the relationship between perceived over-qualification and turnover intention. The results from exploring these components can help to expand the boundary around perceived over-qualification, enrich the theoretical research on human resource management, and help units within the institutional system pay greater attention to and alleviate the problems of employee over-qualification and turnover.

## Theoretical Analysis and Research Hypothesis

### Theoretical Analysis

Over-qualification refers to the level of education, skills, and experience that an individual possesses beyond what is required in their work (Erdogan et al., 2011a; McKee-Ryan and Harvey, 2011; Liu and Mo, 2012). Over-qualification is generally divided into objective and subjective over-qualification (Maynard et al., 2006; Maltarich et al., 2011). Objective over-qualification refers to the notion that employees’ actual ability, knowledge, and skills fully meet the requirements of their work. Subjective over-qualification refers to employees’ perception of their abilities, knowledge and skills that are beyond their actual work requirements. As such, subjective over-qualification is also referred to as perceived over-qualification. Due to the differences in subjective qualification, researchers pay greater attention to subjective over-qualification and its impact on individuals and organizations (Johnson and Johnson, 2000; Edwards, 2008; Maynard and Joseph, 2008).

Although the current research on perceived over-qualification has generated some important findings, the research boundary is constantly expanding. Accordingly, the current study identified several shortcomings in the literature review conducted. First, the existing research pays greater attention to the positive impact of perceived over-qualification on individuals, such as organizational citizenship behavior (Chen and Kao, 2011; Elamin and Tlaiss, 2015; Luksyte and Spitzmueller, 2016) and job satisfaction (Lee et al., 2013; Yang et al., 2015; Dong et al., 2020; García-Mainar and Montuenga-Gómez, 2020). Additionally, a few studies have explored the negative impact of perceived over-qualification on individuals, such as anti-productive behavior (Bolino and Feldman, 2000; Zhang et al., 2016; Schreurs et al., 2020). Second, there is limited research on the relationship between perceived over-qualification and turnover intention. Although it can be speculated that perceived over-qualification may have an impact on employee turnover intention, the relationship between the two remains unclear. When an employee has high perceived over-qualification, the coordination between the employee and their work may be undermined. Employees with high perceived over-qualification are likely to believe their abilities fully qualify them for better jobs and their turnover intention, therefore, is likely high (Sabanciogullari and Dogan, 2015; Ai and Yang, 2016). Liu et al. (2015) believes that perceived over-qualification can trigger both individuals’ cognitive and emotional responses. However, a consensus on the specific influence path and role relationship has not yet been reached.

Self-efficacy is one important construct used to measure individuals’ perceptions of their ability to accomplish tasks (Bandura, 1977; Le and Ji, 2007; Zee and Koomen, 2016). Its effect on measuring individual cognition has been recognized. Self-efficacy refers to an individual’s speculation and judgment regarding whether they have the ability to complete a certain task (Bandura, 1977; Wang et al., 2001). Therefore, the current study explored the influence of employees’ over-qualification perceptions on their turnover intention from the perspective of individual self-efficacy.

In addition to self-efficacy, professional identity is also an important construct that can be used to measure an individual’s emotional state at work (Pratt et al., 2006; Zhang W. et al., 2018). Further, its effect on perceived over-qualification in relation to individual cognition requires further investigation. Professional identity refers to the combination of employees’ positive perceptions, attitudes, and behavioral tendencies toward their profession and internalized professional roles (Baugh et al., 2005; Canrinus et al., 2012; Maor and Hemi, 2021). The development of an employee’s professional identity is based on the collaboration of individuals and colleagues, and is created through interactions between employees and their work environments (Hu and Sang, 2013; Afzal et al., 2019). When an employee’s professional identity is low, it can promote the influence of perceived over-qualification on turnover intention. Alternatively, when professional identity is high, the influence of over-qualifications on turnover intention is suppressed. Therefore, professional identity may affect the relationship between perceived over-qualification and turnover intention.

### Perceived Over-Qualification and Turnover Intention

Perceived over-qualification is developed based on related concepts, such as under-employment and over-education. Perceived over-qualification refers to the notion that employees believe they have more than the required qualifications (e.g., in terms of education level, experience, knowledge, and skills) for the job they are performing (Maynard and Parfyonova, 2013). Perceived over-qualification is an employee’s perception of their own qualifications, skills, and experience (Zhao C. H. et al., 2019; Ma et al., 2020a). By comparing their abilities with their job requirements, employees can determine the degree of alignment between their abilities and the skills needed for the job. According to the “person-job” matching theory, the degree of matching between people and jobs is an important factor that affects employees’ work status and job performance. A high degree of “person-job” matching can make the best use of employees so that such talents can be fully utilized to improve work performance (Onat and Eren, 2020). On the contrary, if the degree of “person-job” matching is low, the dilemma of “over-capability” can arise, which in turn can lead to negative effects.

From a cognitive perspective, employees with high perceived over-qualification believe they are “oversized and under-utilized.” Consequently, they believe it is difficult to obtain development opportunities in their current jobs, which in turn can lead to turnover intention. If an employee’s knowledge and skills are not fully valued, they can feel frustrated and think their current work is preventing them from achieving their ideals (Fine and Nevo, 2008). According to the “person-job” matching theory, whether a job can meet employees’ career desires affects their work attitudes. From an emotional point of view, low-quality “person-job” matching causes employees to work with lower levels of enthusiasm. If a job does not fully utilize an employee’s talents, the employee may feel frustrated and lose interest in their work (Ahmad and Qadir, 2018; Razeena and Bhasi, 2019). Employees with perceived over-qualification may believe the work they are engaged in lacks challenges, they find it challenging to obtain a sense of pleasure in their work, and they find it difficult to satisfy their spiritual pursuit of success in their careers, all of which can result in high turnover intention (Alfes et al., 2016; Zhao L. J. et al., 2019). Therefore, the following hypothesis was developed for the current study:

Hypothesis 1 (H1): There will be a positive relationship between perceived over-qualification and turnover intention.

### Perceived Over-Qualification and Self-Efficacy

Many scholars have explored the negative effects of perceived over-qualification, such as reductions in employee physical and mental health, organizational commitment and job satisfaction, as well as a triggering of turnover intention. According to the “person-job” matching theory, the mismatch, or low-quality match, between employee abilities and job needs can reduce employee enthusiasm and satisfaction, leading to high turnover intentions or counterproductive behavior (Fine and Nevo, 2008). According to the theory of resource preservation, high perceived over-qualification may cause individuals to worry about their own resources being wasted, leading to emotional exhaustion and job burnout. To avoid further depletion of resources, employee distance themselves from work and produce anti-productive behaviors (Cretu and Burcas, 2014; Zhao L. J. et al., 2019). According to the self-regulation theory, self-efficacy is one of the variables most suitable to explain changes in self-regulation (Bandura, 1977; Le and Ji, 2007; Zee and Koomen, 2016). Individuals adjust their current cognition, emotions, and behaviors *via* a conscious self-regulation process and strive to unify them with their own goals, thereby accelerating the realization of those goals (Scheier et al., 2001; Locke et al., 2017). People with high perceived over-qualification may also have a positive view of their over-qualification, believing that they can perform tasks other than their work, or hope to take the initiative to maintain a positive self-image. This ultimately makes them perform more beneficial behaviors for the organization, such as the former proactive behavior and job reshaping.

Self-efficacy refers to the speculation and judgment that an individual makes about whether they have the ability to accomplish a certain task. Bandura (1977) defines self-efficacy as people’s confidence in whether they can use their own skills to complete a task. When those with high perceived over-qualification find that the task is simple and the goal is extremely easy to achieve, driven by the need for self-realization, they will then discover their talents and potential (Hu et al., 2015). Consequently, they may seek broader tasks and evaluate the feasibility of those tasks based on excess knowledge, skills and abilities, all of which may in turn stimulate high self-efficacy. Second, when assessing the resources and constraints required to complete a task, people with high perceived over-qualification generally believe they have more resources (e.g., superb skills, sense of job control) and fewer constraints (e.g., low job requirements and job control) to take on more tasks and responsibilities (Wang et al., 2019). The comparison between these resources and constraints can enhance self-efficacy. Finally, people with high perceived over-qualification are more likely to perform tasks that exceed the requirements of their positions and responsibilities and, thereby, they gain recognition and encouragement from others. People often attribute these positive achievements to their abilities, and this positive internal attribution may have a positive impact on their self-efficacy. Therefore, the following hypothesis was proposed:

Hypothesis 2 (H2): There will be a positive relationship between perceived over-qualification and self-efficacy.

### Self-Efficacy and Turnover Intention

Perceived over-qualification reflects the individual’s subjective perception of the gap between the actual state (the aptitude cannot be fully utilized) and the desired state (the aptitude can be fully utilized). It will cause individuals to have a sense of imbalance, such as “over-capable of underachievers” or “unexpected talents,” and produce certain negative emotions. Existing studies have analyzed the influence of self-efficacy on individual intentions, and some studies have analyzed the effect of self-efficacy on individual behavior (McNatt and Judge, 2008; Yoon and Kim, 2016). According to the theory of planned behavior, individual intention is the direct cause of individual behavior, and an increase in individual turnover intention may promote employee turnover behavior. Therefore, people with a high self-efficacy are more likely to have turnover behavior than those with a low self-efficacy. Self-efficacy affects an individual’s perception of self-ability and goal positioning, as well as the possibility of achieving goals. It is an important psychological resource. Individuals have more confidence in their ability to complete work requirements, and an increase in self-efficacy will further affect employees’ turnover behavior (Jaramillo et al., 2006). Therefore, the following hypothesis is proposed:

Hypothesis 3 (H3): There will be a positive correlation between self-efficacy and turnover intention.

### Effect of Self-Efficacy on the Relationship Between Perceived Over-Qualification and Turnover Intention

Perceived over-qualification affects individuals’ behavior indirectly through two mediating processes: emotional and cognitive responses. Self-efficacy is an important mediating variable in cognitive pathways. When employees have high perceived over-qualification, they are overconfident and their self-efficacy is high. Employees with high perceived over-qualification believe their abilities far exceed the needs of their current job and that they should pursue career development elsewhere. This type of excessive self-awareness and over-confidence enhances self-efficacy and weakens employees’ true understanding of their work, they hope to find better jobs and play to their talents. When people with over-qualification find that the task is simple and extremely easy to achieve, they feel they are over-qualified and can easily complete their tasks using minimal time and energy. Such thoughts can reduce employees’ work enthusiasm and happiness. Moreover, perceived over-qualification can reduce employee enthusiasm, in turn increasing job boredom and turnover intention. Therefore, the following hypothesis was proposed:

Hypothesis 4 (H4): Self-efficacy will have a mediating effect on the relationship between perceived over-qualification and turnover intention.

### Effect of Professional Identity on the Relationship Between Perceived Over-Qualification and Turnover Intention

Professional identity belongs to the content of psychology field, which predominantly measures the consistency of an individual’s professional goals with others’ views of the profession (Pratt et al., 2006). Professional identity includes two dimensions: cognition and emotion (Virta, 2015). When employees build their internal professional identity, they feel the personal value and happiness produced by their profession. Over-qualified groups generally have high work abilities and high expectations for career development (Erdogan et al., 2011b; Zhao C. H. et al., 2019). They expect to use their abilities to do a good job and realize their own value. Therefore, when employees have turnover intention due to their high perceived over-qualification, professional identity can reduce the impact it might have.

The development of teachers’ professional identity is based on collaboration between individuals and professionals, and is constructed through interactions between teachers and their environments (Sachs, 2001; Hong, 2010; Hu and Sang, 2013). Professional identity is related to the research field of positive professional psychology, which refers to the combination of individual positive cognition, experience, attitudes, and behavioral tendencies toward individuals’ professional roles (Beijaard et al., 2004; Wei et al., 2013). Professional identity refers to a mental state in which an individual subjectively accepts and agrees with their own profession and actively devotes themselves to the job. When perceived over-qualification does not match the perception of job demand, coordination between the individual and professional environment may be broken (Maynard and Joseph, 2008; Maltarich et al., 2011). At this time, when employees with high professional identity face an imbalance between their own abilities and professional development needs, based on their love and recognition of their careers, they will actively face their own abilities and professional development needs, attributing more reasons to themselves rather than to the professional environment. This reduces employees’ turnover intention (Wang et al., 2020). For employees with lower professional identity, as they do not agree with their occupations, they are more likely to attribute more reasons to their professional environments. They assume they are very capable but, due to a career that they perceive does not fit them, they do not fully utilize their talents, in turn accelerating their turnover intention (Korthagen, 2004). Therefore, the following hypothesis was proposed:

Hypothesis 5 (H5): Professional identity will have a counter-regulatory effect on the relationship between perceived over-qualification and turnover intention.

### Effect of Professional Identity on the Relationship Between Self-Efficacy and Turnover Intention

Professional identity can have a positive effect on individual career development and job engagement either directly or indirectly, and can have a negative effect on job burnout, depression, anxiety, and turnover intention (Korthagen, 2004; Wang and Du, 2014; Zhang W. et al., 2018). Studies indicate that professional identity may affect the relationship between self-efficacy and turnover intention. The author believes professional identity, as a motivating trait, has become one of the most important psychological factors for individuals, where it can deeply affect employees’ feelings about their careers and behaviors. Employees with high professional identity actively adjust their daily work status to obtain greater satisfaction. Further, owing to their love for their career, employees reduce the impact of self-efficacy on turnover intention (Wong and Cheng, 2020) and will work more enthusiastically and actively. When employees’ professional identity is low, the influence of self-efficacy on turnover intention is intensified. Therefore, the following hypothesis was proposed:

Hypothesis 6 (H6): Professional identity will play a moderating role in the relationship between self-efficacy and turnover intention.

**FIGURE 1:**
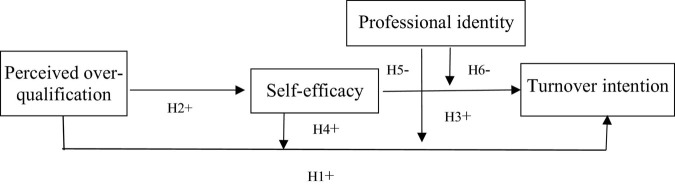
Theoretical model.

Figure 1 outlines the theoretical model, which shows the relationship between perceived over-qualification and turnover intention.

## Methodology

### Measures

Perceived over-qualification (POQ) scale was developed by Maynard and Parfyonova (2013) and contain nine items. The questionnaire uses a 5-point Likert scale ranging from 1 (*strongly disagree*) to 5 (*strongly agree*). The Cronbach’s alpha for the perceived over-qualification scale is 0.806. The self-efficacy (SE) scale with 10 items was compiled by Schwarzer and Jerusalem (1995). This study used the Chinese version of the self-efficacy scale translated and revised by Chen et al. (2001) and Wang et al. (2001). The scale uses a 5-point Likert scale ranging from 1 (*strongly disagree*) to 5 (*strongly agree*). The Cronbach’s alpha for this self-efficacy scale is 0.902. This research draws on the professional identity (PI) scale compiled by psychologists Jou et al. (2013) and Wei et al. (2013). This scale is well-known in the professional identity research field. The scale contains three dimensions: professional values, professional belonging, and role values (with a combined total of 18 items). The scale uses a 5-point Likert scale ranging from 1 (*strongly disagree*) to 5 (*strongly agree*). The Cronbach’s alpha for the professional self-efficacy scale is 0.950. This study refers to the turnover intention scale compiled and used by Lobene and Meade (2013). This study used the Chinese version of the turnover intention scale translated and revised by Zhang K. L. et al. (2018) for the Chinese context. The Cronbach’s alpha of the turnover intention scale is 0.927. Based on existing research, it is thought employee characteristics could impact the study’s findings. Therefore, demographic characteristics such as gender, age, education, years employed and income level were included as control variables.

### Data Collection and Analysis

The current study recruited primary school teachers as participants and predominantly investigated the relationship between primary school teachers’ perceived over-qualification, self-efficacy, and turnover intention. Selecting one or more sites to obtain the desired amount of sample data is common practice. The educational resources in Fujian Province are at a medium level, which can represent the average level of teacher’ perceived over-qualifications in China. This province also provided a strong opportunity for the convenient collection of survey data. An author has accumulated many years of work experience as an elementary school teacher; therefore, they were suited to conducting the research. Many school leaders, teachers and education departments in China assisted with the completion of this study.

The convenience sampling method proposed by Ferreira et al. (2019) was primarily used to conduct the survey of teachers from July to October 2020. To collect a bigger sample, this study also used the popular snowball sampling approach (Yoon and Kim, 2016; Moon et al., 2017), which makes it possible to reach large numbers of potential participants across a broad spectrum of sample distribution. Prior to the study being conducted, the survey team employed performed convenience sampling of teachers across different schools. The investigator first determined whether they were the research objects *via* a brief communication (e.g., face-to-face, phone call or email). Next, the investigator explained the aims of the study to the potential participants and asked whether they would like to fill out the survey. At the very beginning of the questionnaire, the rules for filling in the questionnaire will be given to ensure that participants know how to fill out the questionnaire. A total of 500 surveys were distributed and 402 surveys were returned. Eight surveys were excluded from the study based on missing data and, thus, a total of 396 valid surveys were obtained for the study (this equated to a valid response rate of 79.2 percent). Table 1 presents the demographic characteristics of the survey sample.

**TABLE 1:** Frequency and percentage of the sample.

**Variable**	**Options**	**Frequency**	**Percentage**	**Variable**	**Options**	**Frequency**	**Percentage**
Gender	Male	69	17.4	Age	Under 30 years old	143	36.1
	Female	327	82.6		31–40 years old	125	31.6
Education	Undergraduate	375	94.7		41–50 years old	110	27.8
	Postgraduate	21	5.3		Over 50 years old	18	4.5
Year	Under 5 years	80	20.2	Income	Under 3,000 yuan	9	2.3
	6–10 years	103	26.0		3,000–5,000 yuan	113	28.5
	11–15 years	85	21.5		5,000–7,000 yuan	138	34.8
	16–20 years	68	17.2		7,000–10,000 yuan	99	25
	Over 20 years	60	15.2		Over 10,000 yuan	37	9.3

### Reliability and Validity Test

Table 2 shows the results of the reliability and validity tests. This study used the Harman single factor test method proposed by Podsakoff et al. (2000) to test the common method deviation using SPSS 26.0. The results revealed that the unrotated maximum factor variance explanation rate was 29.34 percent, which was lower than the 50 percent discriminant value, indicating that there was no serious homology deviation between the scales. The results showed that the KMO value of the perceived over-qualification, self-efficacy, professional identity, and turnover intention scales were 0.817, 0.908, 0.933, and 0.841, respectively. All the scales passed the KMO and Bartlett tests.

**TABLE 2:** Reliability and validity test results.

**Variable**	**KMO**	**Chi-square**	**Df**	**Sig.**	**Cumulative contribution rate%**	**Cronbach’s alpha**
Perceived over-qualification	0.817	1,040.682	36	0.000	54.73	0.809
Self-efficacy	0.908	2,301.202	45	0.000	65.383	0.904
Professional identity	0.933	5,275.769	153	0.000	67.374	0.950
Turnover intention	0.841	2,177.32	28	0.000	61.476	0.909

The scale instruments used in this study are well-established and recognized by researchers. To further test the validity of the scales, confirmatory factor analysis (CFA) was conducted based on general academic norms (Hu and Bentler, 1999). The analysis results of the revised model revealed an acceptable fit: perception over-qualification scale (χ2/Df = 1.350, NFI = 0.990, RFI = 0.954, CFI = 0.997, and RMSEA = 0.030), self-efficacy scale (χ2/Df = 2.496, NFI = 0.988, RFI = 0.952, CFI = 0.993, and RMSEA = 0.062), professional identity (χ2/Df = 3.387, NFI = 0.933, RFI = 0.903, CFI = 0.951, and RMSEA = 0.078), and turnover intention scale (χ2/Df = 3.595, NFI = 0.993, RFI = 0.954, CFI = 995, and RMSEA = 0.081).

## Results

### Correlation Analysis

Table 3 presents the results of the correlation analysis conducted in the current study. Perceived over-qualification was positively correlated with self-efficacy (*r* = 0.146) and turnover intention (*r* = 0.154), and self-efficacy was positively correlated with turnover intention (*r* = 0.211). There was a positive correlation between professional identity and turnover intention (*r* = −0.488). The results further revealed a correlation between perceived over-qualification, self-efficacy, professional identity, and turnover intention.

**TABLE 3:** Means, standard deviations, and correlations.

**Variable**	**1**	**2**	**3**	**4**	**5**	**6**
1. Turnover intention	1	0.154**	0.211**	−0.488**	0.100*	−0.001
2. Perceived over- qualification	0.154**	1	0.146**	−0.123*	0.191**	0.052*
3. Self-efficacy	0.211**	0.146**	1	−0.028	0.038	0.099*
4. Professional identity	−0.488**	−0.123*	−0.028	1	−0.043	0.044
5. Education	0.100*	0.191**	0.038	−0.043	1	0.167**
6. Income	−0.001	0.052*	0.099*	0.044	0.167**	1
Mean	2.899	3.310	2.394	3.888	3.050	3.110
Std.	0.701	0.729	0.803	0.714	0.224	0.996

* and ** indicate a significant correlation at the 0.05 and 0.01 levels, respectively.

### Perceived Over-Qualification

Table 4 presents the results of the two-way ANOVA. Through correlation analysis, it was found that education and income were related to perceived over-qualification. Therefore, this study further explored the differences in perceived over-qualification and turnover intention among employees with different education and income levels.

**TABLE 4:** Results of two-way ANOVA analysis.

**Source**	**Variable**	**Type III sum of squares**	**Df**	**Mean square**	**F**	**Sig.**	**Eta**
Calibration model	Perceived over-qualification	17.725^a^	9	1.969	4.310	0.000	0.091
	Turnover intention	37.928^b^	9	4.214	7.493	0.000	0.149
Intercept	Perceived over-qualification	590.109	1	590.109	1,291.334	0.000	0.770
	Turnover intention	361.764	1	361.764	643.217	0.000	0.625
Education	Perceived over-qualification	3.053	1	3.053	6.681	0.010	0.017
	Turnover intention	0.341	1	0.341	0.607	0.437	0.002
Income	Perceived over-qualification	8.054	4	2.013	4.406	0.002	0.044
	Turnover intention	14.916	4	3.729	6.630	0.000	0.064
Education × Income	Perceived over-qualification	5.013	4	1.253	2.743	0.028	0.028
	Turnover intention	4.221	4	1.055	1.876	0.114	0.019
Error	Perceived over-qualification	176.393	386	0.457			
	Turnover intention	217.097	386	0.562			
Total	Perceived over-qualification	3,522.802	396				
	Turnover intention	2,525.078	396				

*^*a*^R Squared = 0.072 (Adjusted R Squared = 0.051); ^*b*^R Squared = 0.062 (Adjusted R Squared = 0.040).*

The results of the two-way ANOVA revealed that teachers with different levels of education had significant differences in perceived over-qualification (*p* < 0.01). Teachers with different income levels had significant differences in perceived over-qualification (*p* < 0.01) and turnover intention (*p* < 0.01). Additionally, an interaction effect of education and income on perceived over-qualification was revealed (*p* < 0.05).

**FIGURE 2:**
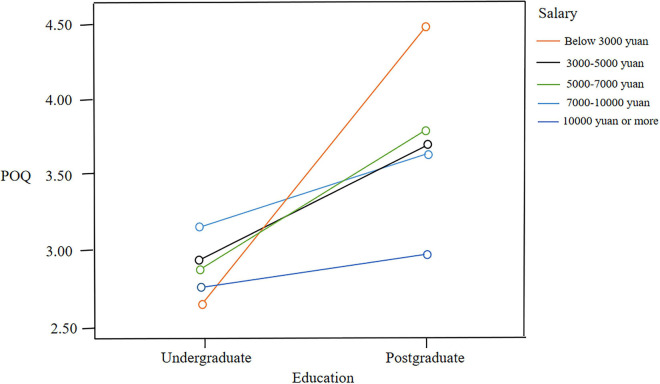
The interaction of education and income on POQ.

Table 5 shows the results of the one-way ANOVA used in the current study. The results revealed that the perceived over-qualification of teachers with high education was significantly higher than that of teachers with low education (*p* < 0.01), and the turnover intention of teachers with high education was significantly higher than that of teachers with low education (*p* < 0.05). Additionally, the results showed that the perceived over-qualification of high-income teachers was significantly greater than that of middle-income teachers. When teachers’ income reached a certain level, their turnover intention began to increase. Figure 2 shows the interaction effect of education and income on POQ.

**TABLE 5:** Results of one-way ANOVA analysis.

**Variable**		**Options**	**Mean**	**Std.**	**Sig**
Education	POQ	Undergraduate (1)	2.868	0.687	0.002
		Postgraduate (2)	3.465	0.771	
	TI	Undergraduate (1)	2.370	0.767	0.043
		Postgraduate (2)	2.732	1.186	
Salary	POQ	Below 3,000 yuan (1)	2.888	0.673	0.039 (5 > 4,3,2)
		3,000–5,000 yuan (2)	2.913	0.633	
		5,000–7,000 yuan (3)	2.882	0.719	
		7,000–10,000 yuan (4)	2.792	0.706	
		10,000 yuan or more (5)	3.219	0.788	
	TI	Below 3,000 yuan (1)	2.611	0.740	0.150 (5 > 4)
		3,000–5,000 yuan (2)	2.375	0.757	
		5,000–7,000 yuan (3)	2.428	0.776	
		7,000–10,000 yuan (4)	2.251	0.813	
		10,000 yuan or more (5)	2.604	0.924	

### Hierarchical Regression Analysis

Table 6 shows the results of the hierarchical regression analysis employed for the current study. The dependent variable of M1, M3, and M4 was turnover intention, and the dependent variable of M2 was self-efficacy. The M2 model revealed that perceived over-qualification was significantly positively correlated with self-efficacy (β = 0.136). Thus, H2 holds. The M3 model revealed there was a significant positive correlation between perceived over-qualification and turnover intention (β = 0.134) and, thus, H1 holds. The M4 model showed that there was a significant positive correlation between self-efficacy and turnover intention (β = 0.182), supporting H3. Additionally, perceived over-qualification was significantly positively correlated with turnover intention (β = 0.111). Finally, self-efficacy was found to partially mediate the relationship between perceived over-qualification and turnover intention—that is, perceived over-qualification was found to affect employees’ turnover intention through self-efficacy. Therefore, H4 is supported.

**TABLE 6:** Results for the main effect and mediating role.

**Variable**	**M1**	**M2**	**M3**	**M4**
Gender	−0.052 (−1.037)	−0.046 (−0.917)	−0.092* (−1.858)	−0.029 (−0.586)
Age	0.02 (0.195)	0.008 (0.073)	0.106 (1.017)	−0.012 (−0.114)
Education	0.073 (1.395)	0.047 (0.899)	−0.021 (−0.391)	0.051 (0.985)
Year	−0.171 (−1.598)	−0.157 (−1.479)	−0.204* (−1.924)	−0.12 (−1.142)
Income	0.048 (0.841)	0.045 (0.79)	0.135** (2.395)	0.02 (0.358)
Perceived over-qualification		0.136*** (2.688)	0.134*** (2.649)	0.111** (2.222)
Self-efficacy				0.182*** (3.636)
*F*	2.515	3.334	3.419	4.836
Adj.R2	0.019	0.034	0.035	0.064
△R2	0.031	0.018	0.017	0.031

*, **, and *** indicate a significant correlation at the 0.1, 0.05, and 0.01 levels, respectively.

Table 7 shows the results of the moderating role. The dependent variable for M5–M8 was turnover intention. The M6 model revealed a significant negative correlation between perceived over-qualification × professional identity and turnover intention (β = −0.885), indicating professional identity reversely moderated the relationship between perceived over-qualification and turnover intention. Thus, H4 is supported. The M8 model showed that self-efficacy × professional identity was significantly negatively correlated with turnover intention (β = −0.707), indicating professional identity negative moderated the relationship between self-efficacy and turnover intention. Thus, H6 is supported.

**TABLE 7:** Results for the moderation role.

**Variable**	**M5**	**M6**	**M7**	**M8**
Gender	−0.016 (−0.356)	0.001 (0.008)	−0.001 (−0.018)	−0.003 (−0.079)
Age	0.048 (0.523)	0.062 (0.686)	0.034 (0.377)	0.055 (0.618)
Education	0.035 (0.765)	0.038 (0.823)	0.049 (1.104)	0.043 (0.968)
Year	−0.176* (−1.886)	−0.183** (−1.981)	−0.144 (−1.566)	−0.171* (−1.866)
Income	0.064 (1.296)	0.068 (1.392)	0.041 (0.833)	0.035 (0.713)
Perceived over-qualification	0.08* (1.786)	0.797*** (3.704)		
Self-efficacy			0.185*** (4.256)	0.715*** (3.887)
Professional identity	−0.472*** (−10.708)	0.119 (0.664)	−0.477*** (−11.094)	−0.01 (−0.063)
Perceived over-qualification × professional identity		−0.885*** (−3.406)		
Self-efficacy × professional identity				−0.707*** (−2.963)
*F*	20.073	19.493	22.953	21.585
Adj.R2	0.253	0.272	0.280	0.294
△R2	0.217	0.021	0.262	0.016

*, **, and *** indicate a significant correlation at the 0.1, 0.05, and 0.01 levels, respectively.

### Bootstrap Analysis

**FIGURE 3:**
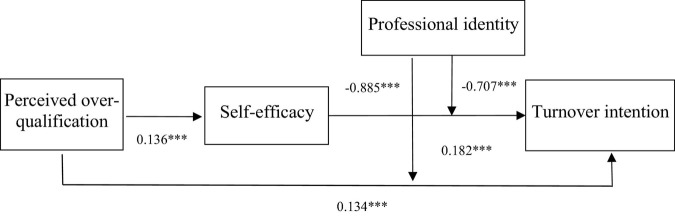
Verified theoretical model. *, **, and *** indicate a significant correlation at the 0.1, 0.05, and 0.01 levels, respectively.

Table 8 shows the results of the bootstrap analysis for the self-test conducted in the current study. Hayes and Preacher (2014) highlighted that the bootstrap method performs better when multivariate relationships are analyzed. Therefore, the current study used the Bootstrap method for the self-test. The results revealed that perceived over-qualification was significantly positively correlated with turnover intention and self-efficacy, hypothesis 1 and 2 is verified. Self-efficacy was significantly positively correlated with turnover intention, hypothesis 3 is verified. The mediation analysis revealed a direct effect coefficient of perceived over-qualification on turnover intention of 0.2159 (LLCI = 0.0806, ULCI = 0.3513), and this was significant, as the confidence interval did not include zero. The indirect effect coefficient of perceived over-qualification on turnover intention was found to be 0.0413 (LLCI = 0.0013, ULCI = 0.0954). The confidence interval for this effect did not contain zero, indicating that perceived over-qualification affected turnover intention through self-efficacy. Hypothesis 4 is verified. The results of the moderation analysis revealed that the moderating effect coefficient of professional identity on perceived over-qualification and turnover intention was −0.205 (LLCI = −0.3213, ULCI = −0.0797). The confidence interval for this analysis did not contain zero, thus revealing a significant result. The moderating effect coefficient of professional identity on self-efficacy and turnover intention was found to be −0.1505 (LLCI = −0.2517, ULCI = −0.0492), and the confidence interval did not include zero. This shows that professional identity had a moderating effect on perceived over-qualification and the relationship between self-efficacy and turnover intention. The results of the bootstrap test also verified the hypothesis 5. Figure 3 and Table 9 show the results of all hypothesis test.

**TABLE 8:** Result for self-test analysis.

**Variable**	**Effect**	**Se**	**T**	**P**	**LLCI**	**ULCI**
Constant	–1.1347	1.1353	–0.9995	0.3182	–3.367	1.0975
Gender	0.026	0.0898	0.2901	0.7719	–0.1504	0.2025
Age	0.0567	0.0782	0.7243	0.4693	–0.0972	0.2105
Education	0.1267	0.1591	0.7964	0.4263	–0.1861	0.4394
Year	–0.0840	0.0438	–1.9185	0.0558	–0.1700	0.0021
Income	0.0314	0.0388	0.8088	0.4191	–0.0449	0.1076
Perceived over-qualification	0.8286	0.2406	3.4442	0.0006	0.3556	1.3016
Self-efficacy	0.7570	0.2004	3.7772	0.0002	0.3629	1.1510
Professional identity	–0.6008	–0.2605	–2.306	0.0216	–0.0885	–1.1130
Perceived over-qualification × professional identity	–0.2005	0.0614	–3.2644	0.0012	–0.3213	–0.0797
Self-efficacy × professional identity	–0.1505	0.0515	–2.9217	0.0037	–0.2517	–0.0492

**TABLE 9:** Result for self-test analysis.

**Hypothesis**	**Path coefficient**	**Significance**
Hypothesis H1: There is a positive relationship between perceived over-qualification and turnover intention.	0.134 (*P* < 0.01)	Yes
Hypothesis H2: There is a positive relationship between perceived over-qualification and self-efficacy.	0.136 (*P* < 0.01)	Yes
Hypothesis H3: There is a positive correlation between self-efficacy and turnover intention.	0.182 (*P* < 0.01)	Yes
Hypothesis H4: Self-efficacy has a mediating effect on the relationship between perceived over-qualification and turnover intention.	0.111 (*P* < 0.05)	Yes
Hypothesis H6: Professional identity has a counter-regulatory effect on the relationship between perceived over-qualification and turnover intention.	−0.885 (*P* < 0.01)	Yes
Hypothesis H5: Professional identity has a counter-regulatory effect on the relationship between self-efficacy and turnover intention.	−0.707 (*P* < 0.01)	Yes

## Discussion

This study used an ANOVA to characterize employees’ perceived over-qualification based on 396 participant surveys from China. It then used hierarchical regression analysis and the bootstrap method to analyze the relationship between perceived over-qualification and turnover intention. Specifically, this study explored the influence of individual perceived over-qualification on turnover intention by introducing self-efficacy as a mediating variable. Simultaneously, a moderating variable, professional identity, was added to measure the relationship between perceived over-qualification, self-identity and turnover intention.

The main findings of the study are as follows. First, the results revealed that the average value of teachers’ perceived over-qualification was 3.310, which is indeed higher than that of employees in previous studies (e.g., Simon et al., 2018; Zhao C. H. et al., 2019; Zhao L. J. et al., 2019). Corporate employees with higher qualifications may have higher salaries in the market economy. Teachers have higher education and qualifications, but their qualifications and salaries do not match market prices. Additionally, teachers’ resignation is restricted through China’s educational system and, thus, teachers’ resignation is not an easy process. Therefore, their perceived over-qualification may be higher than that of corporate employees.

Second, the perceived over-qualification of teachers with high education was significantly higher than that of teachers with low education, and the turnover intention of teachers with high education was significantly higher than that of teachers with low education. Additionally, the perceived over-qualification of high-income teachers was significantly greater than that of middle-income teachers. When teachers’ income reached a certain level, their turnover intention began to increase. Education and income revealed an interactive effect on perceived over-qualification. Specifically, when teachers’ income reaches a certain level, they are more willing to pursue personal ideals and values. Further, the higher their education, the higher their value mission may be. Therefore, the higher a teacher’s income and education levels are, the stronger their perceived over-qualification and the greater their turnover intention.

Third, perceived over-qualification was significantly positively correlated with turnover intention. When teacher’ perceived over-qualification is high, so too is their turnover intention. According to the “person-job” matching theory (Mobley et al., 1978), when an individual’s personal characteristics are inconsistent with the professional environment in which they work, many problems can arise, including those associated with their turnover behavior (Tims et al., 2016; Ding et al., 2019). From a cognitive perspective, employees with high perceived over-qualification believe they are “oversized and under-utilized.” This makes it difficult for them to obtain development opportunities in their current jobs, and this can lead to the development of turnover intention. From an emotional point of view, low-quality “person-job” matching can cause employees to work with lower levels of enthusiasm. Employees with perceived over-qualification may believe the work they are engaged in lacks challenges. This can lead to dissatisfaction with respect to their spiritual pursuit of career success and, in turn, result in high turnover intention.

Fourth, self-efficacy revealed a mediating role on the relationship between perceived over-qualification and turnover intention, that is, perceived over-qualification can affect turnover intention by enhancing employees’ self-efficacy. Teachers with high perceived over-qualification are more likely to perform tasks that exceed the requirements of their positions and responsibilities, which can lead to greater self-efficacy. Teachers with high self-efficacy believe their tasks are simple and easy to accomplish. This can create certain negative emotions that can promote their turnover intention.

Finally, professional identity had a moderating role in the relationship between perceived over-qualification and turnover intention. For employees with high professional identity, such identity can inhibit perceived over-qualification from promoting turnover intention. High professional identity among teachers can reduce the impact of self-efficacy on turnover intention based on their love for their career, that is, under such conditions, they are more likely to actively and enthusiastically work.

### Theoretical Contribution

The current study makes several theoretical contributions to the cognitive psychology and teacher management field. First, perceived over-qualification and turnover intention constitute important research content in terms of organizational behavior. Research on their relationship enriches and expands the theoretical literature on organizational management. Existing studies on perceived over-qualification have predominantly focused on corporate employees, ignoring cohorts in institutional settings, such as teachers. Teachers are often highly educated and qualified, and their perceived over-qualification may therefore be higher than that of corporate employees. Indeed, the results of the current study revealed that teachers perceived over-qualification was higher than that recorded for employees in previous studies (Simon et al., 2018; Zhao C. H. et al., 2019). However, relatively few studies on the perceived over-qualification of groups such as teachers exist. Therefore, this study recruited primary school teachers as research participants, which helps to expand the boundary around the theoretical knowledge on perceived over-qualification. This study also applied the “person-job” matching theory to the field of education, which helps to enrich our current knowledge on individual behavior and organizational management.

Second, this study took the prominent “person-job” mismatching and over-qualification problems of primary school teachers as the starting point for analysis of the resignation problem. Specifically, it explored the impact of primary school teachers’ perceived over-qualification on turnover intention. The study found that perceived over-qualification had a significant positive impact on turnover intention. The results revealed another antecedent variable of turnover intention, enriched the influence boundary of perceived over-qualification, and expanded and responded to the research of scholars such as Zimmerman (2010) and Lobene and Meade (2013). The results obtained in the current study build on the existing theoretical foundation in this research area and help to explore in greater detail the related research on perceived over-qualification. They also serve to find another inducement for the teacher’s resignation problem.

Third, based on knowledge of the cognitive path, this study analyzed the influence mechanism of perceived over-qualification on turnover intention. Previous studies have found that self-efficacy has a significant positive effect on turnover intention. This conclusion responds to the research of Zhang W. et al. (2018) and verifies the relationship between self-efficacy and turnover intention. The current study found that self-efficacy had a mediating effect on the relationship between perceived over-qualification and turnover intention, opening up understandings around the effect of the perceived over-qualification cognitive path (the “black box”) on turnover intention and testing the transmission mechanism of self-efficacy between perceived over-qualification and turnover intention. Responding to the research suggestions of Hu et al. (2015) and Dong et al. (2020), these findings broaden our understandings of another mediation path concerning perceived over-qualification and its outcome variables. Perceived over-qualification was found to significantly positively affect self-efficacy. This conclusion supports notions that perceived over-qualification helps to promote self-efficacy.

Finally, the current study also found that professional identity can moderate the relationship between perceived over-qualification and turnover intention. Organizations should endeavor to reduce the negative effects of over-qualification perception on employees by cultivating employees’ professional identities. These findings reinforce the important role of professional identity, increase the boundary conditions for the negative impact of over-qualification perception, and helped to reveal another inhibitory factor for the negative impact of perceived over-qualification (Wu et al., 2015; Li et al., 2020). Further, they expand the theory of organizational management and also provide opportunity for further investigation into employee’ perceived over-qualification.

### Practical Implication

First, the current study found that teachers with different education and income levels significantly differed in their perceived over-qualification, and education and income had an interactive effect on perceived over-qualification. When teachers have high levels of education, the psychological pride and self-confidence induced by high levels of education can also lead to excessively high ability perception. When employees are on high incomes, they pay less attention to their income and pursue personal value and happiness. Additionally, when this is the case, employees are full of confidence in their abilities, and their perceived over-qualification is also high. Perceived over-qualification can cause an imbalance between employee needs and job offers, in turn leading to negative emotion and sabotage behavior among employees. Therefore, schools should focus on employing those individuals who are highly-educated and are on high incomes, as well as take intervention measures in advance to guide highly-educated and over-qualified employees so that their talents and values can be maximally utilized. This in turn can help to reduce teachers’ negative emotion and turnover intention. For example, schools need to take guiding measures so that management can prevent the loss of talent. Simultaneously and while providing a high income, schools must also prevent negative behavior among over-qualified teachers. By setting an example, organizations should encourage employees to work actively while also creating friendly and positive work environments. Organizations should also mobilize employees’ enthusiasm through a reward system and career guidance. The current study helps to provide a reference for optimizing organizational management and improving organizational effectiveness.

Second, perceived over-qualification had a positive effect on turnover intention. Therefore, schools need to pay attention to the problem of turnover intention caused by high perceived over-qualification. With continuous improvements in the qualifications of elementary school teachers, the problems caused by high perceived over-qualification continue to increase. Schools should pay more attention to elementary school teachers and implement effective measures to alleviate the problems associated with teacher’ perceived over-qualification and cognitive biases. This can help to improve organizational management. Through active guidance and by strengthening organizational management, schools can prevent perceived over-qualification from becoming the main reason teachers decide to leave.

It was found perceived over-qualification affects turnover intention through the enhancement of self-efficacy. Therefore, schools should not only focus on employees’ reactions based on their emotional changes but also cultivate deeper understandings of employees’ perceptions of their abilities and professional needs. Schools can use vocational training, organizational learning, and publicity to strengthen employees’ understanding of their job positions and career development needs. This can help employees develop a clearer understanding of their abilities and working environment, and can also help to reduce employees’ cognitive biases.

Finally, the current study found that professional identity regulates the impact of perceived over-qualification on turnover intention. Therefore, schools can strengthen teachers’ awareness of their occupation through vocational training. Schools should strengthen the implementation of social responsibility, publicize the outstanding deeds of the organization, establish a good organizational image, and cultivate employees’ professional identity. At the same time, organizations must also create a good working environment and a standardized promotion and rewards system in order to win the recognition of teachers and strengthen the positive guiding role organizational culture can play.

## Conclusion

Unlike previous studies focusing on perceived over-qualification, the current study analyzed the relationship between perceived over-qualification and turnover intention from the cognitive perspective (e.g., self-efficacy). The results revealed that perceived over-qualification was associated with turnover intention *via* the mediating role of self-efficacy. Additionally, professional identity was found to play a moderating role in the relationship between perceived over-qualification and turnover intention. Through analyzing differences in perceived over-qualification, this study provides a lead for developing a deeper understanding on the teachers’ resignation problem. This study marks a major step forward in studying the predictive pathways of the effects of perceived over-qualification on teachers’ turnover intention. This research also expands the influence boundary of perceived over-qualification and provides theoretical support for organizational management.

The study contains several limitations. First, considering individual differences, cultural background and the nature of an individual’s employment may have an impact on perceived over-qualification and turnover intention. The current study, however, only focused on factors such as participant gender, education, and income level. Additionally, this study focused on the impact of over-qualification perception on turnover intention with respect to the cognitive path and, therefore, did not analyze the impact of the emotional path. Further, Ma et al. (2020b) and Li et al. (2021) proposed that perceived over-qualification affects career planning and knowledge dissemination. Therefore, the antecedent and outcome variables of over-qualification from the perspective of knowledge hiding and career distress could be explored further in future research.

## Data Availability Statement

The raw data supporting the conclusions of this article will be made available by the authors, without undue reservation.

## Ethics Statement

Ethical review and approval was not required for the study on human participants in accordance with the local legislation and institutional requirements. The patients/participants provided their written informed consent to participate in this study.

## Author Contributions

GC conceived of the initial idea, designed the study, and drafted the manuscript. YS collected and analyzed the data and drafted the manuscript. YT and GC revised and proofread the manuscript. All authors contributed to the article and approved the submitted version.

## Conflict of Interest

The authors declare that the research was conducted in the absence of any commercial or financial relationships that could be construed as a potential conflict of interest.

## Publisher’s Note

All claims expressed in this article are solely those of the authors and do not necessarily represent those of their affiliated organizations, or those of the publisher, the editors and the reviewers. Any product that may be evaluated in this article, or claim that may be made by its manufacturer, is not guaranteed or endorsed by the publisher.
